# Brain Protein Expression Profile Confirms the Protective Effect of the ACTH_(4–7)_PGP Peptide (Semax) in a Rat Model of Cerebral Ischemia–Reperfusion

**DOI:** 10.3390/ijms22126179

**Published:** 2021-06-08

**Authors:** Olga Yu. Sudarkina, Ivan B. Filippenkov, Vasily V. Stavchansky, Alina E. Denisova, Vadim V. Yuzhakov, Larisa E. Sevan’kaeva, Liya V. Valieva, Julia A. Remizova, Veronika G. Dmitrieva, Leonid V. Gubsky, Nikolai F. Myasoedov, Svetlana A. Limborska, Lyudmila V. Dergunova

**Affiliations:** 1 Institute of Molecular Genetics of National Research Center Kurchatov Institute, Kurchatov Sq. 2, 123182 Moscow, Russia; filippenkov@img.msk.ru (I.B.F.); bacbac@yandex.ru (V.V.S.); lia97@mail.ru (L.V.V.); utoshkautoshka@gmail.com (J.A.R.); veronuska@mail.ru (V.G.D.); nfm@img.msk.ru (N.F.M.); limbor@img.msk.ru (S.A.L.); lvd@img.msk.ru (L.V.D.); 2 Department of Neurology, Neurosurgery and Medical Genetics, Pirogov Russian National Research Medical University, Ostrovitianov str. 1, 117997 Moscow, Russia; dalina543@gmail.com (A.E.D.); gubskii@mail.ru (L.V.G.); 3 A. Tsyb Medical Radiological Research Center–Branch of the National Medical Research Radiological Center of the Ministry of Health of the Russian Federation, Zhukov st. 10, 249031 Obninsk, Russia; yuzh_vad@mail.ru (V.V.Y.); larisa.sevankaeva@mail.ru (L.E.S.); 4 Federal Center for the Brain and Neurotechnologies, Federal Biomedical Agency, Ostrovitianov str. 1, Building 10, 117997 Moscow, Russia

**Keywords:** ACTH_(4–7)_PGP (Semax), ischemic stroke, tMCAO, gene and protein expression profile, immunodetection, real-time RT-PCR, spreading depression

## Abstract

The Semax (Met-Glu-His-Phe-Pro-Gly-Pro) peptide is a synthetic melanocortin derivative that is used in the treatment of ischemic stroke. Previously, studies of the molecular mechanisms underlying the actions of Semax using models of cerebral ischemia in rats showed that the peptide enhanced the transcription of neurotrophins and their receptors and modulated the expression of genes involved in the immune response. A genome-wide RNA-Seq analysis revealed that, in the rat transient middle cerebral artery occlusion (tMCAO) model, Semax suppressed the expression of inflammatory genes and activated the expression of neurotransmitter genes. Here, we aimed to evaluate the effect of Semax in this model via the brain expression profiling of key proteins involved in inflammation and cell death processes (MMP-9, c-Fos, and JNK), as well as neuroprotection and recovery (CREB) in stroke. At 24 h after tMCAO, we observed the upregulation of active CREB in subcortical structures, including the focus of the ischemic damage; downregulation of MMP-9 and c-Fos in the adjacent frontoparietal cortex; and downregulation of active JNK in both tissues under the action of Semax. Moreover, a regulatory network was constructed. In conclusion, the suppression of inflammatory and cell death processes and the activation of recovery may contribute to the neuroprotective action of Semax at both the transcriptome and protein levels.

## 1. Introduction

For many years, ischemic stroke has been one of the main causes of mortality and disability among the populations of many developed countries. The search for new therapeutic strategies for stroke treatment remains an urgent problem. A significant contribution to the solution can be made by a detailed study of the mechanisms of action of drugs that have a neuroprotective effect against cerebral ischemia. Peptides of the melanocortin family are actively involved in the functioning of the central nervous system because they act as neuroprotective agents [1]. 

The neuroprotective effects of melanocortins in acute and chronic neurodegenerative conditions are manifested through the antagonism of excitotoxic, inflammatory, and apoptotic reactions, which are the main damaging mechanisms associated with ischemia, as well as through neurogenic effects [2,3,4,5]. Melanocortins are a family of peptide hormones formed from a common precursor, the proopiomelanocortin molecule. Melanocortins includes the group of melanocyte-stimulating hormones (α-, β-, and γ-MSH) and adrenocorticotropic hormone (ACTH) [6]. 

In particular, α-MSH, the amino acid sequence of which coincides completely with the site of amino acids 1–13 of ACTH, exhibits noticeable anti-inflammatory, neurogenic, and neuroprotective effects in models of experimental ischemia [5,7]. The synthetic peptide Semax is a hybrid molecule; its N-terminus contains a fragment of ACTH (4–7)-Met-Glu-His-Phe, which coincides with a similar site of α-MSH, whereas its C-terminus is stabilized by the addition of the biogenic tripeptide Pro-Gly-Pro, which affords greater metabolic stability. 

Without hormonal and toxic side effects, the peptide increases the rate and extent of recovery of neurological functions [8], reduces the severity of neurological deficits, and prolongs the survival of animals with ischemic stroke [9]. Being a synthetic derivative of natural melanocortins, Semax exhibits several of the important neuroprotective properties of α-MSH: it potentiates dopamine and serotoninergic transmission in the striatum [10], exerts neurotrophic effects in primary neuronal cultures [11], and promotes the survival of neurons under conditions of glutamate neurotoxicity stress [12]. 

The study of the molecular mechanisms underlying the actions of Semax using models of cerebral ischemia in rats showed that the peptide acts on the brain transcriptome: it enhances the transcription of neurotrophins and their receptors [13,14], significantly affects the expression of genes associated with the processes of the immune response [15,16], suppresses the activation of the expression of genes involved in inflammation, and prevents the decrease in the expression of genes associated with neurotransmission [17]. 

The aim of the present study was to evaluate the effect of Semax on the expression of several key proteins involved in inflammation and cell death processes, as well as neuroprotection and recovery in stroke. In this work, we used a rat model of transient occlusion of the middle cerebral artery (tMCAO) to investigate the effect of Semax on the expression level of the protein products of several genes that are the target of the α-MSH neuroprotective action in the subcortical structures of the brain that contained the focus of ischemic injury, as well as in the adjacent frontoparietal cortex at 24 h after occlusion. 

As the role of these proteins in the pathogenesis of stroke is well known, this may contribute to the understanding of the molecular mechanisms of action of this peptide. These proteins include matrix metalloproteinase 9 (MMP-9), which is activated during inflammation and may serve as a prognostic indicator of stroke severity [18]; c-Jun N-terminal kinases (JNKs), the activation of which is involved in inflammation and apoptosis [19]; the transcriptional factor c-Fos, which plays a significant role in postischemic inflammation and cell death [20]; and the cyclic adenosine monophosphate (cAMP) response element-binding (CREB) transcription factor, which prevents neuronal damage and is involved in the neuroprotective effect of α-MSH [4,21]. 

We found that, under the influence of Semax, the expression profiles of several of the studied proteins in the subcortical structures and the cortex of ischemic rats differed markedly. However, the general effect of the drug in both tissues was to compensate for the level of expression of most of the proteins that were impaired by ischemia.

## 2. Results

### 2.1. Morphology of Brain Tissues in the Experimental Groups

**Figure 1:**
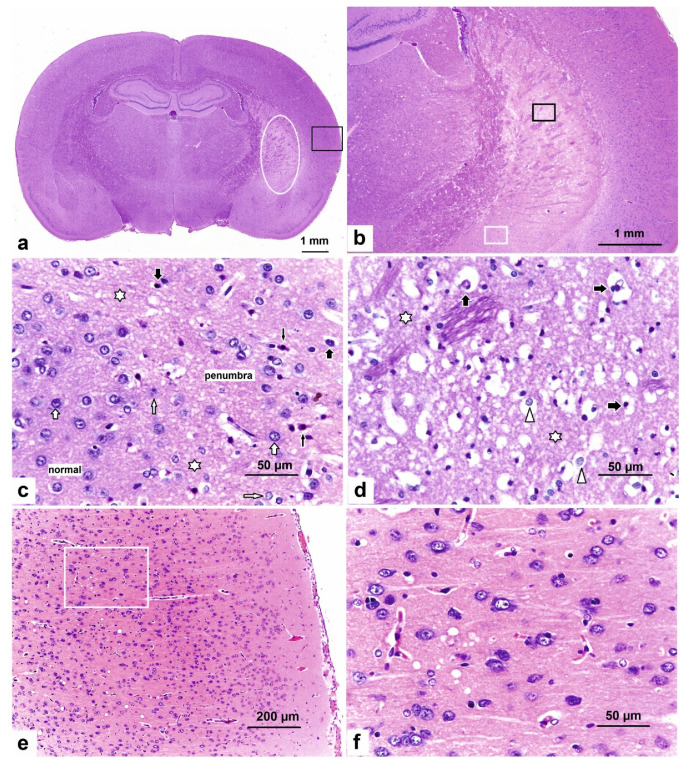
Photomicrographs of H&E-stained sections of the rat brain after 24 h in tMCAO model conditions. (**a**) Coronal rat brain section at the level of −2.5 mm from the bregma. The oval shape indicates the damaged area involving the caudoputamen nucleus of the right hemisphere. The rectangle indicates the secondary somatosensory cortex. (**b**) The zone of ischemic injury of the caudoputamen indicated within panel (**a**) is shown at a higher magnification. (**c**) Area of panel (**b**) marked with a white rectangle. The change zone: from normal to ischemic-injured (penumbra) tissue; intact neurons (thick white arrows); decrease of nuclear basophilia in the neurons (thin white arrows); ischemic damage to neurons, with pyknotic nuclei and pericellular edema (thick black arrows); red neurons (thin black arrows); and vesiculation of the neuropile (white asterisks). (**d**) Area of panel (**b**) marked with a black rectangle. The ischemic core: vacuolation with edema of the neuropile (white asterisks); swollen neurons (white triangles); necrotic cells (thick black arrows); and absence of intact neurons. (**e**) The area of panel (**a**) marked with a rectangle. Normal cytoarchitecture of the secondary somatosensory cortex. (**f**) The area of panel (**e**) marked with a white rectangle. The inner pyramidal (V; ganglionic) layer. The perikarya of large pyramidal cells are filled with basophilic substances.

In standard coronal sections from experimental animals that were stained with hematoxylin and eosin (H&E), an ischemic lesion of the brain tissue in the form of “enlightenment” was found in the lateral zones of the subcortical region of the right brain hemisphere of rats at 24 h after tMCAO. The developmental regions of the subcortical lesion zone were observed in the caudoputamen region, from +1.7 to −3.3 mm from the bregma. At the level of the hippocampus, the infarction zone exhibited an elongated shape in the dorsoventral direction and captured most of the caudoputamen lateral region (Figure 1a,b), with visualization of the penumbra (Figure 1c) and the deep-ischemic core (Figure 1d).

Concomitantly, the area of the developing ischemic damage covered the caudoputamen to the outer capsule. Microscopic examination revealed a drop in the lumen of the capillaries and their emptying, pronounced perivascular edema, sparsity and bleaching of the neuropil caused by edema and vesiculation, and the appearance of numerous hyperchromic neurons with pericellular edema.

According to pathomorphological criteria, a fraction of the neurons located within the outer boundary zone of the lesion were normal; many neurons exhibited hypoxic damage (reduction of basophilic chromatin in the nuclei, edema, and homogenization of the cytoplasm); and death (nuclear pyknosis with increased cytoplasmic eosinophilia (red neurons), destruction of nuclei, and cytoplasmic lysis) (Figure 1c). Vacuolation with edema of neuropils, as well as swollen and necrotic neurons, was observed in the ischemic core (Figure 1d). There were no neurons with a normal morphology in the deep-ischemic zone.

There were no microscopic signs of ischemic damage to the nervous tissue in the cortex of the right hemisphere. The cytoarchitecture of the cerebral cortex along the layers of neurons was without features (Figure 1e). In the neocortex, neuropils had a fine-grained, fine-fiber base. The vessels of the microcirculatory bed were moderately full-blooded. The basophilic substance was clearly visualized in the perikaryon of neurons (Figure 1f).

### 2.2. Changes in the Levels of pJNK, pCREB, MMP-9, and c-Fos in the Subcortical Structures and Cortex of the Rat Brain under the Conditions of Ischemia–Reperfusion

Using the Western blot method, we evaluated the changes in the total content of the active forms of JNK, namely phospho-(Thr183/Tyr185)-SAPK/JNK 54 kDa and 46 kDa (pJNK) and active phospho-(Ser133)-CREB (pCREB), as well as in the level of the MMP-9 and c-Fos proteins in the subcortical structures and cortex of rats at 24 h after the onset of occlusion in the groups of animals with “ischemia–reperfusion” (IR) relative to the animals of the group with “sham operation” (SH), as well as under the effect of Semax in ischemia (IS) relative to the IR group. The β-actin protein was used as the internal control.

**Figure 2:**
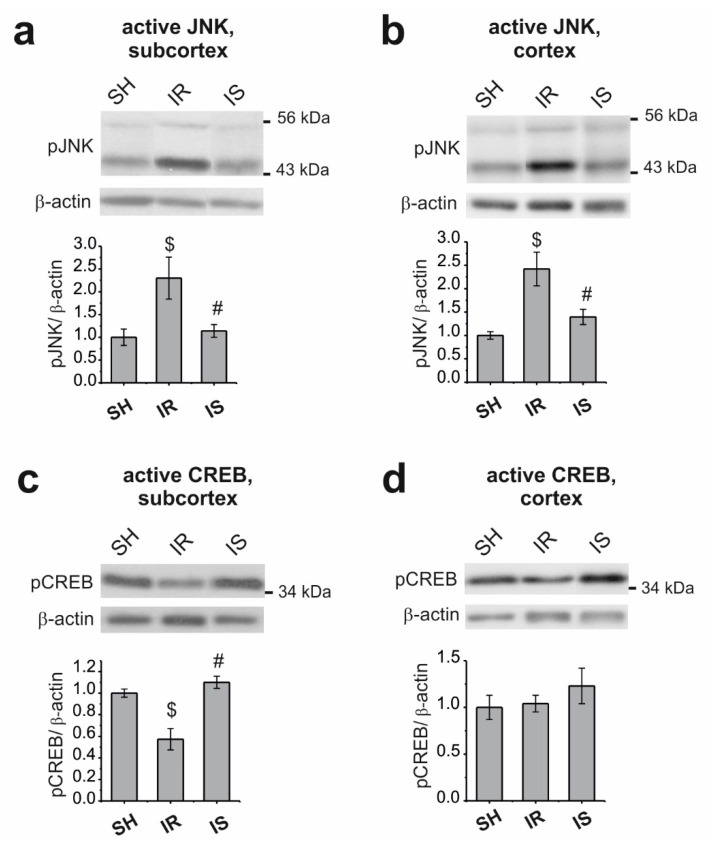
Analysis of changes in the level of the pJNK and pCREB proteins during ischemia and Semax treatment in the subcortical structures and frontoparietal cortex of rats at 24 h after tMCAO. Immunoblot of pJNK (**a**,**b**) and pCREB (**c**,**d**) content in the subcortex and cortex. The upper part of the figures shows a typical immunoblot for each group. The histograms report the relative protein content in the group, which was normalized to that of β-actin; the protein content in the SH group is taken as “1”. Statistical significance is indicated above the bars as $ *p* < 0.05 and # *p* < 0.05, IR versus SH and IS versus IR, respectively. The data are presented as the mean ± standard error of the mean (S.E.M.) (n = 5–7 animals per group).

The total content of pJNK was increased by more than two-fold in the subcortex (Figure 2a) and cerebral cortex (Figure 2b) of the IR relative to the SH group; in turn, these levels were decreased by more than 1.5-fold after treatment with Semax.

The level of pCREB was decreased by more than 1.4-fold in the subcortex during ischemia, whereas Semax increased its levels by more than 1.5-fold (Figure 2c). The level of pCREB did not change in the cerebral cortex of rats in the IR group; however, treatment with Semax also increased the levels of this protein, although this change was not statistically significant (Figure 2d).

**Figure 3:**
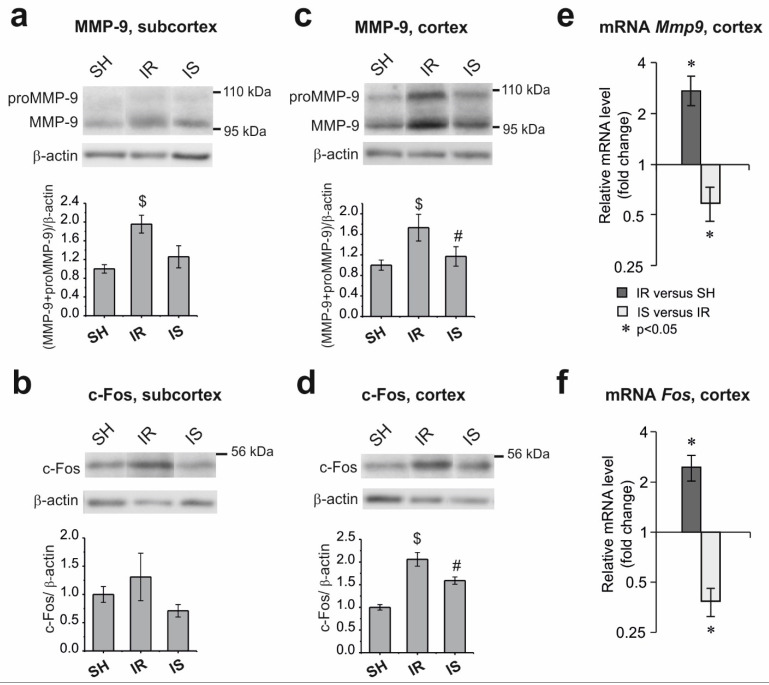
Analysis of the changes in the expression of the MMP-9 and c-Fos proteins, as well as the *Mmp9* and *Fos* mRNA, during ischemia and Semax treatment in rat brain structures at 24 h after tMCAO. Immunoblot analysis of MMP-9 and c-Fos content in the subcortex (**a**,**b**) and frontoparietal cortex (**c**,**d**). The upper part of the figures shows a typical immunoblot for each group. The histograms report the relative protein content in the group, which was normalized to that of β-actin; the protein content in the SH group is taken as “1.” The statistical significance is indicated above the bars as *, $ *p* < 0.05 and # *p* < 0.05, IR versus SH and IS versus IR, respectively. Changes in the level of the *Mmp9* (**e**) and *Fos* (**f**) mRNA expression in the rat frontoparietal cortex are measured in the IR versus SH and IS versus IR groups. The mRNA level of genes in the groups is normalized to the mRNA level of the *Gapdh* gene.

According to the Western blot analysis, in the subcortical structures of the brain of animals in the IR group, the total content of the MMP-9 protein was increased by approximately 1.7-fold relative to the SH group (Figure 3a). A decrease in the level of MMP-9 was noted after Semax administration, although this difference was not significant. The changes in the c-Fos protein content in the subcortex were not statistically significant in the studied groups (Figure 3b).

In the cerebral cortex of the animals in the IR group, the levels of the MMP-9 (Figure 3c) and c-Fos (Figure 3d) proteins were significantly increased by about two-fold compared with the SH group, whereas the introduction of Semax led to a statistically significant decrease in their levels by more than 1.5-fold (IS versus IR).

### 2.3. Changes in the mRNA Level of the Mmp9 and Fos Genes in the Frontoparietal Cortical Area Adjacent to the Ischemic Damage Zone

Real-time PCR was used to analyze the changes in the mRNA level of the *Mmp9* and *Fos* genes in the frontoparietal cortex in the IR relative to the SH group, as well as in the IS relative to the IR group. The mRNA level of the *Gapdh* and *Rpl3* genes was used as the internal control. We found that, under the conditions of ischemia–reperfusion, the levels of the *Mmp9* and *Fos* mRNAs were increased by more than two-fold in the frontoparietal cortex of the rats adjacent to the damaged area (Figure 3e,f). Concomitantly, Semax had the opposite effect of downregulating both genes. Thus, in rats in the IS group compared with animals in the IR group, the level of *Mmp9* mRNA was decreased by approximately 1.7-fold (Figure 3d), and the level of the *Fos* mRNA was decreased by 2.6-fold (Figure 3e).

### 2.4. Analysis of the Involvement of the JNK, CREB, MMP-9, and c-Fos Proteins in the Signaling Pathways That Were Activated during Ischemia and the Semax Treatment

To analyze the signaling pathways that involved the JNK, CREB, MMP-9, and c-Fos proteins during ischemia and the Semax treatment, we used the results of genome-wide data clustering that were published previously by our research group [17,22]. The use of the Cytoscape 3.7.0 program (Institute for Systems Biology, Seattle, WA, USA) [23] based on the data of the association of differentially expressed genes with functional annotations allowed us to illustrate the participation of pJNK and pCREB in the signaling pathways that exhibited changes in their activities under ischemic conditions, as well as the effect of Semax in the subcortical structures of the brain at 24 h after occlusion. 

**Figure 4:**
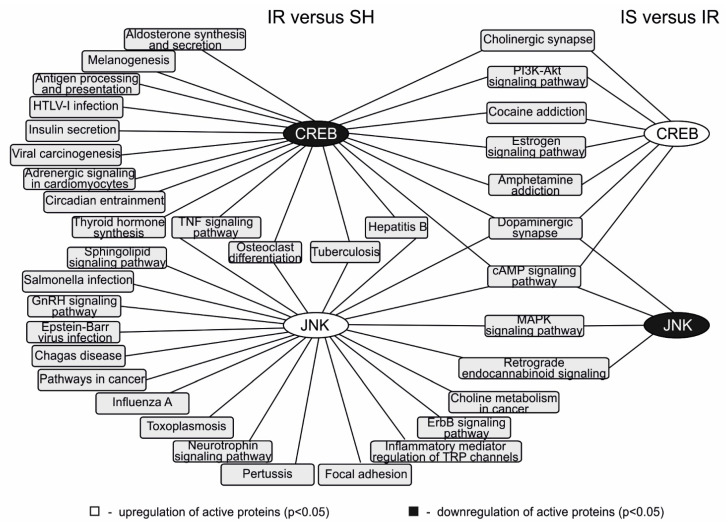
Scheme of JNK and CREB participation in the signaling pathways identified by us previously [17,22] and associated with ischemia–reperfusion and Semax action in the subcortical structures of the brain at 24 h after tMCAO. The network was constructed using the Cytoscape 3.7.0 program. The oval blocks at the nodes represent proteins. The gray rectangular boxes represent signaling pathways. The lines connecting the oval and rectangular blocks indicate the participation of the protein in the signaling pathway.

The network obtained (Figure 4) showed that the downregulation of pCREB during ischemia was associated with the modulation of the activity of 20 signaling pathways, including antigen processing and presentation and the TNF, PI3K-Akt, and cAMP signaling pathways, among others. In contrast, the administration of Semax in the conditions of IR upregulated pCREB led to a change in the activity of seven signaling pathways (dopaminergic and cholinergic synapses, PI3K-Akt, cAMP signaling pathways, etc.). It is important to note that all of these pathways were also modulated by ischemia–reperfusion compared with the sham operation. 

Concomitantly, the upregulation of pJNK during ischemia was associated with the modulation of the activity of 22 signaling pathways, including focal adhesion and the neurotrophin, sphingolipid, and mitogen-activated protein kinases (MAPK) signaling pathways. In contrast, the administration of Semax under ischemic conditions downregulated pJNK, which led to a change in the activity of four signaling pathways (retrograde endocannabinoid signaling, dopaminergic synapses, and the MAPK and cAMP signaling pathways). The activity of all of these signaling pathways was modulated by ischemia–reperfusion in the damaged area compared with the corresponding brain regions in the SH group (Figure 4).

As we did not record the changes in the level of the MMP-9 and c-Fos proteins in subcortical structures during ischemia and Semax treatment, we were not able to illustrate the spectrum of signaling pathways associated with these proteins. However, we found that c-Fos was involved in the functioning of four signaling pathways (dopaminergic and cholinergic synapses, amphetamine addiction, and the cAMP signaling pathway), whereas MMP-9 was involved in estrogen signaling pathway functioning. Moreover, these pathways might be modulated by the activity of CREB or JNK under the action of Semax after the establishment of tMCAO conditions.

## 3. Discussion

The peptide drug Semax has a pronounced neuroprotective effect in the treatment of cerebrovascular insufficiency and acute cerebral stroke [8,24,25,26]. However, the molecular mechanism underlying the action of this peptide has not been established and is being actively investigated. Previously, under the conditions of the tMCAO model, we used genome-wide RNA-Seq analysis to identify large-scale changes in genome activity under the effect of Semax at 24 h after occlusion in the subcortical structures of the rat brain where the ischemic zone was located. The peptide upregulated many genes that showed decreased expression after ischemia, and in turn, downregulated genes that were activated by ischemia [17]. 

In this work, to further investigate the mechanisms underlying the neuroprotective action of Semax under the conditions of the tMCAO model, the expression of the pJNK, MMP-9, c-Fos, and pCREB proteins, which are actively involved in the pathogenesis of ischemic stroke, was analyzed by immunodetection in the subcortical structures and the frontoparietal cortex of the rat ipsilateral hemisphere.

pJNK, MMP-9, and c-Fos are important factors in the progression of ischemic stroke. JNK belongs to the family of MAPK that respond to stress, including stress caused by reactive oxygen species, excitotoxicity, and inflammatory cytokines. Research found that stress stimuli cause the rapid and long-term activation of the JNKs involved in ischemic cell death and neuroinflammation, while their inhibition following experimental ischemia causes a pronounced neuroprotective effect [27,28]. Phosphorylated JNK (pJNK) alters the activity of many proteins, including the activation of the transcription factors c-JUN, ATF2, and SP-1 [29].

The activation of the MMP-9 protein, which disrupts the stability of the blood–brain barrier, promotes edema and stroke progression [30]. It has also been shown that MMP-9 expression could be induced by proinflammatory cytokines through the NF-κB and MAPK signaling pathways [31,32].

The transcription factor c-Fos plays a significant role in post-ischemic inflammation [33]. As part of the AP-1 transcription factor, c-Fos activates many genes involved in ischemic damage and inflammation, such as *IL*-6, *TNF*-α, *CCL2*, matrix metalloproteinase genes, and genes involved in apoptosis. However, by activating late effector genes involved in post-stroke regeneration (*NGF*, *bFGF*, etc.), c-Fos is involved in the prevention of neuronal death in ischemic conditions [34]. There is also evidence that the ischemic induction of c-Fos is mediated primarily by an increase in intracellular calcium, followed by the activation of extracellular signal-regulated (ERK) kinases [20].

According to immunodetection data, the content of the MMP-9 protein was increased in the subcortical structures and cortex of rats under the conditions of ischemia–reperfusion. We did not observe an effect of Semax on the level of the MMP-9 protein in the subcortex, where the damaged area was localized. However, in the cortex, the peptide downregulated the *Mmp9* gene both at the mRNA and protein levels (Figure 3). Thus, a protective effect of Semax directed at the expression of the *Mmp9* gene was not detected in the subcortex, which included a large area of damage, but was observed in the cortex adjacent to the infarction area, which contained morphologically intact but likely functionally deficient cells.

Under the experimental conditions used here, and in response to ischemic damage, an increase in the c-Fos protein content was observed in the rat brain cortex, and a tendency for its upregulation was detected in the subcortex (Figure 3). There is evidence that ischemia rapidly activates the expression of the *c-Fos* mRNA and protein in 2-h rat tMCAO models across the entire ipsilateral hemisphere, with the maximum induction observed after 1 h of reperfusion, followed by a decrease in expression to the peri-infarction areas and the maintenance of a high expression level along the infarction border at 24 h after tMCAO. 

Concomitantly, starting at 4 h after reperfusion, the expression of the c-Fos protein is not detected in the infarction nucleus [35]. The absence of a statistically significant change in the level of the c-Fos protein in the subcortical structures in our experiment may be attributed to bidirectional changes in its expression in the peri-infarction areas and the necrotic core, which could lead to a significant scatter of values within animal groups. Semax did not affect the expression of c-Fos in the subcortical structures of the brain. 

However, in the area of the frontoparietal cortex, which did not contain the damaged area, the levels of the *c-Fos* mRNA and protein under the action of the peptide were noticeably reduced after their ischemic activation and were practically indistinguishable from the levels detected in sham-operated animals. The decrease in the expression of the *c-Fos* gene under the action of Semax in the cortex is in agreement with the known suppression of glutamate excitotoxicity by the peptide [12] and may indicate a decrease in ischemic cell death.

The content of pJNK was increased both in the subcortical region of the rat brain and in the cortical area adjacent to it in animals of the IR group. These data are in good agreement with the results of studies that showed an increase in JNK phosphorylation and in the activity of the JNK signaling pathway in the brains of rodents after ischemia [36]. Under the action of Semax, we found a decrease in the level of JNK kinases in the two brain structures under study, which may be associated with the activation of compensatory mechanisms under the action of the peptide both in the regions of the brain containing the focus of the ischemic damage and in the tissues adjacent to them.

Unlike MMP-9, pJNK, and c-Fos, the phosphorylated form of the CREB transcription factor (pCREB) regulates the transcriptional programs of synaptic plasticity, neurogenesis, and neuronal survival in the brain [37]. CREB also functions as a key hub in the neurotrophin, antiapoptotic, and antioxidant gene network [38]. A decrease in CREB expression and activation levels was found in the ischemic stroke focus, whereas an increase in these parameters was detected in the peri-infarct area [39]. 

Many drugs, such as cerebrolysin and resveratrol, which have a neuroprotective effect in stroke, also increase the level of active CREB in ischemic brain tissues [40,41]. Moreover, melanocortin receptors, which mediate the neuroprotective effect of α-MSH, activate CREB [42]. In our experiment, a decrease in the level of pCREB was detected under the conditions of ischemia–reperfusion in the subcortical structures of the brain at 24 h after the onset of occlusion, which was completely rescued by Semax. It should be noted that, in the studied area of the cortex, neither ischemia–reperfusion nor Semax had a significant effect on the pCREB content.

Thus, the changes in the expression of the pJNK, pCREB, MMP-9, and c-Fos proteins in the brain of rats subjected to ischemia–reperfusion detected under the action of Semax confirm the protective effect of the peptide, which had been previously revealed at the transcriptomic level. The observation that the effect of Semax on the pCREB content was manifested only in the subcortex, whereas that of the MMP-9 and c-Fos proteins was observed in the cortex and that of pJNK was detected in the cortex and subcortex, apparently reflects both significant differences and similarities in the molecular and cellular mechanisms underlying the pathogenesis of damage, and neuroprotection in the zones of infarction and areas of the brain outside ischemia.

It is well known that ischemic injury results in the formation of an infarct nucleus surrounded by an ischemic penumbra. According to the results of our histological examination at 24 h after tMCAO, the infarction nucleus and the penumbra zone were localized in the subcortical region of the brain, while no gross pathological changes were detected in the frontoparietal cortex. 

Figure 1 shows that the infarct nucleus includes completely necrotic nerve tissue with the destruction of all elements of the neuropile and dead “pyknomorphic” neurons, whereas the neurons detected in the penumbra zone exhibited signs of both ischemic damage and a lack of pronounced pathological changes. It is believed that the effects of neuroprotective drugs on the viable cells surrounding the focus of ischemic damage and with a high risk of death provide the possibility of reversing brain damage. It can be assumed that, in the subcortical structures of the brain, the manifestation of the protective effect of Semax occurs via changes in the levels of pJNK and pCREB under the action of the peptide. 

In turn, the role of pJNK and pCREB under the action of Semax may be associated with their participation in the modulation of neurotransmission and inflammation signaling pathway activity in subcortical structures (Figure 4). We have previously shown that, in the subcortical structures of the brain containing the focus of ischemic damage, Semax initiates a large-scale neurotransmitter and anti-inflammatory response that counteracts ischemia–reperfusion [17]. 

Figure 4 shows that both pJNK and pCREB are involved in the functioning of a variety of signaling pathways associated with inflammation and nerve signal transmission in the subcortex, both under the effect of ischemia and after Semax administration. It is interesting to note that the signaling pathways in which each of the proteins is involved exhibit almost no overlap. Only 6 out of the 36 identified signaling pathways are simultaneously associated with the activity of both pJNK and pCREB in the subcortical structures of the brain containing the zone of ischemic injury. At the same time, only two out of nine pathways are associated with the activity of both proteins under the action of Semax. 

Thus, pJNK and pCREB are likely to be involved in the functioning of signaling pathways in the area of ischemic injury, to a large extent, independently of each other. We showed that pJNK was upregulated in this region of the brain during ischemia, followed by its downregulation after the administration of Semax; in contrast, pCREB was downregulated during ischemia and upregulated under the effect of Semax. Thus, the activity of pJNK and pCREB may be an important element in the mechanism underlying the neuroprotective action of Semax, including explaining the multiple compensatory effects of the peptide in the zone of damage after ischemia–reperfusion.

A limitation of our study is the analysis of the expression of only four proteins; the genome-wide proteomic analysis may reveal some additional proteins involved in the response to Semax administration during ischemia. A detailed study of the expression of mRNA and proteins will definitely confirm the features of the functioning of genes under the influence of the peptide during ischemia in various brain structures.

The role of the cortical cells lying outside the area of visible damage in the molecular and cellular mechanisms of ischemic damage and the possible protective effect of drugs remains an open question. It is known that, together with excitotoxicity, oxidative stress, and inflammation, a significant role is played by the spreading depression (SD) in the pathophysiology of ischemic stroke [43]. This is a temporary wave of almost complete depolarization of neurons and glia that is associated with large-scale transmembrane ionic and water shifts caused by massive damage and that occurs at the border between the ischemic zone and the penumbra and spreads to intact areas. 

The SD is an important factor in secondary injury in stroke and leads to profound transcriptomic, metabolic, and hemodynamic effects [44,45,46]. It can be assumed that the change in the activity of pJNK, as well as the changes in the *Mmp9* and *Fos* genes at the mRNA and protein levels detected here in the ipsilateral region of the rat frontoparietal cortex under IR conditions are largely attributable to the SD effect. Semax may weaken the secondary damage caused by the destructive effects of SD by reducing the expression of the *Mmp9* and *Fos* genes in the cortex and has a neuroprotective effect in this area of the brain.

## 4. Materials and Methods

### 4.1. Animals

White 2-month-old male rats of the Wistar line (weight, 200–250 g) were obtained from the Experimental Radiology sector in A. Tsyb Medical Radiological Research Center, Obninsk, Russian Federation. The animals were maintained on a 12-h light/dark cycle at a temperature of 22–24 °C, with free access to food and water.

### 4.2. The tMCAO Model and Semax Administration

The tMCAO model was induced by endovascular occlusion of the right middle cerebral artery using a monofilament (Doccol Corporation, Sharon, MA, USA) for 90 min and was performed under the control of magnetic resonance imaging (MRI), as described previously [22]. Ninety-minute tMCAO in rodents is an adequate duration of occlusion and has been used in numerous studies of the molecular mechanisms of ischemia–reperfusion (IR) damage and the effects of neuroprotective agents on the infarct area [47,48,49,50]. 

Animals that had been subjected to 1.5-h occlusion and subsequent reperfusion were treated with intraperitoneal injections of Semax at a dose of 100 μg/kg (IS) or saline (0.9% NaCl) (IR) immediately after occlusion and at 1.5- and 5-h after the onset of reperfusion. Sham-operated controls (SH) were treated with saline at the same time points. The rats were decapitated 24 h after tMCAO. The experiment design is schematically shown in Supplementary Figure S1.

In our conditions, according to an MRI investigation of the ischemic animals, the damaged area was localized in the right subcortex (subcortical localization) or in the subcortex plus the cortex. To obtain the most representative results, animals with a subcortical localization of the damaged area were used exclusively, for the immunodetection of proteins in the rat brain and analysis of gene expression using real-time reverse transcription polymerase chain reaction (RT–PCR) in the cortex. Supplementary Figure S2 shows the MRI of ischemic foci with a subcortical localization at 24 h after tMCAO.

### 4.3. RNA Isolation

Tissues were placed in RNAlater solution (Ambion, Austin, TX, USA) for 24 h at 0 °C and then stored at –70 °C. The total RNA from the subcortex, was isolated using TRI Reagent (MRC, Cincinnati, OH, USA) and acid guanidinium thiocyanate–phenol–chloroform extraction [51]. The isolated RNA was treated with deoxyribonuclease I (DNase I) (Thermo Fisher Scientific Baltics UAB, Vilnius, Lithuania) in the presence of RiboLock ribonuclease (RNase) inhibitor (Thermo Fisher Scientific Baltics UAB, Vilnius, Lithuania), according to the manufacturer’s recommended protocol. Deproteinization was performed using a 1:1 phenol:chloroform mixture. The isolated RNA was precipitated with sodium acetate (3.0 M, pH 5.2) and ethanol. The RNA integrity was checked using capillary electrophoresis (Experion, BioRad, Hercules, CA, USA). The RNA integrity number (RIN) was at least 9.0.

### 4.4. cDNA Synthesis

cDNA synthesis was conducted in 20 μL of reaction mixture containing 2 mg of RNA using the reagents of a RevertAid First Strand cDNA Synthesis Kit (Thermo Fisher Scientific Baltics UAB, Vilnius, Lithuania) in accordance with the manufacturer’s instructions. Oligo (dT)_18_ primers were used to analyze the mRNA.

### 4.5. Real-Time RT–PCR

The 25 μL polymerase chain reaction (PCR) mixture contained 2 μL of 0.2× reverse transcriptase reaction sample, forward and reverse primers (5 pmol each), 5 μL of 5× reaction mixture (Evrogen Joint Stock Company, Moscow, Russia) including PCR buffer, Taq DNA polymerase, deoxyribonucleoside triphosphates (dNTP), and the intercalating dye SYBR Green I. Primers specific to the genes studied were selected using OLIGO Primer Analysis Software version 6.31 (Molecular Biology Insights Inc., Colorado Springs, CO, USA) and were synthesized by the Evrogen Joint Stock Company (Supplementary Table S1). The amplification of cDNA was performed using a StepOnePlus Real-Time PCR System (Applied Biosystems, Foster City, CA, USA) in the following mode: stage 1 (denaturation), 95 °C, 10 min; stage 2 (amplification with fluorescence measured), 95 °C, 15 s; 65 °C, 25 s; and 72 °C, 35 s (40 cycles).

### 4.6. Data Analysis of Real-Time RT–PCR and Statistics

Two reference genes, *Gapdh* and *Rpl3,* were used to normalize the cDNA samples [52]. Calculations were performed using BestKeeper, version 1 [53] and Relative Expression Software Tool (REST) 2005 software (gene-quantification, Freising-Weihenstephan, Bavaria, Germany) [54]. The manual at the site ‘REST.-gene-quantification.info’) was used to evaluate the expression target genes relative to the expression levels of the reference genes. 

The values were calculated as Ef^Ct(ref)^/Ef^Ct(tar)^, where Ef is the PCR efficiency, Ct(tar) is the average threshold cycle (Ct) of the target gene, Ct(ref) is the average Ct of the reference gene, and Ef^Ct(ref)^ is the geometric average Ef^Ct^ of the reference genes. The PCR efficiencies were assessed using the amplification of a series of standard dilutions of cDNAs and computed using REST software [54]. The efficiency values for all PCR reactions were in the range of 1.90 to 2.01 (Supplementary Table S1). At least six animals were included in each comparison group (n ≥ 6). When comparing data groups, statistically significant differences were considered with the probability *p* < 0.05. Additional calculations were performed using Microsoft Excel (Microsoft Office 2010, Microsoft, Redmond, WA, USA).

### 4.7. Protein Level Analysis by Immunodetection (Western Blotting)

The total protein content was isolated from brain tissues simultaneously with a total RNA extraction using the TRI Reagent (MRC, USA) according to the manufacturer’s protocol. Protein pellets were precipitated with acetone from the organic phase and dissolved in 2% sodium dodecylsulfate (SDS). Protein concentrations were determined using a Lowry-based protein assay (RC DC protein assay, Bio-Rad, Hercules, CA, USA). Samples of 12–45 µg were separated using SDS–PAGE according to the method of Laemmli [55] and transferred by wet electrotransfer to a PVDF membrane (Immun-Blot PVDF Membrane; pore size, 0.2 μm; Bio-Rad, Hercules, CA, USA). 

The membranes were blocked and incubated overnight with primary antibodies against phospho-(Thr183/Tyr185)-SAPK/JNK (Cell Signaling, Danvers, MA, USA), phospho-(Ser133)-CREB (MyBioSource, San Diego, CA, USA), MMP9 (BioVision Inc., Milpitas, CA, USA), c-Fos (MyBioSource), or β-actin (Thermo Fisher Scientific, Rockford, IL, USA). Subsequently, the membranes were incubated with a specific peroxidase-conjugated secondary antibody (Thermo Fisher Scientific, Waltham, MA, USA) for 2 h at 4 °C. 

The signals were visualized using the chemiluminescent peroxidase substrate Lumigen ECL Ultra (Beckman Coulter, Southfield, MI, USA) and imaged using a ChemiDoc MP Visualization System (Bio-Rad, Hercules, CA, USA) or Hyperfilm ECL (Cytiva, formerly GE Healthcare, Marlborough, MA, USA). The protein signals were quantified with scanning densitometry using the ImageQuant 5.2 software (Molecular Dynamics, Sunnyvale, CA, USA). The protein abundance was normalized to that of β-actin.

The protein levels in the rat groups (n = 5–7 animals per group) were expressed as the mean ± standard error of the mean (S.E.M.). Student’s t test was used to evaluate the differences between groups. The significance was set at *p* < 0.05. The most representative blots are shown in Figure 2 and Figure 3. The complete raw images of the blots are provided in Supplementary Figure S3.

### 4.8. Histological Examination of the Rat Brains

The preparation of materials for studies with a histotopographic notch of the brain and frontal orientation of tissue blocks for their subsequent microtomy in the caudorostral direction was as reported previously [22].

Sections of tissue samples of rat brains collected at 24 h after occlusion (n = 4) with a thickness of 5–6 µm obtained through 0.5–1 mm on a microtome (Leica RM2235, Germany) were stained with H&E (BioVitrum, Saint-Petersburg, Russia) after dewaxing. Histological specimens were examined under a microscope (Leica DM 1000), and micrographs were acquired using a digital camera (Leica ICC50 HD). Morphological analysis was performed with an allowance for normal and pathological central nervous system variants [56,57,58]. Stereotactic mapping of the damaged zones and accurate determination of the level of sections was performed according to an atlas of the rat brain [59].

### 4.9. Analysis of pJNK and pCREB Involvement in Signaling Pathways

The participation of the active forms of JNK and CREB in the signaling pathways that were previously identified by our group [17,22] was analyzed, and a network of connections was constructed using Cytoscape 3.7.0. Additional data processing was carried out using Microsoft Excel.

## 5. Conclusions

Our data revealed the effect of Semax on the activity of several proteins that play an essential role in the processes of damage and recovery of brain cells under the pathological effects of ischemia. Concomitantly, the results obtained here allow us to note that, at 24 h after the occlusion of the middle cerebral artery, Semax exerted various effects in the subcortical structures of the brain that contained the focus of ischemic damage, as well as in the adjacent area of the frontoparietal cortex. 

In the subcortex, Semax inhibited pJNK and activated pCREB without affecting the level of MMP-9 and c-Fos; however, in the cortex (which contained the area adjacent to the ischemic core), Semax downregulated pJNK, MMP-9, and c-Fos, and did not change the levels of pCREB. Nevertheless, the general effect of the drug on both brain tissues occurred via its ability to compensate for the expression profiles of these proteins, which were disturbed by ischemia. It should be noted that CREB activation and JNK inhibition are involved in the anti-inflammatory and neuroprotective action of melanocortins in models of ischemic and inflammatory processes in the brain [4,60]. 

The modulation of the activity of the pJNK, pCREB, MMP-9, and c-Fos proteins in various brain regions of animals revealed here may contribute significantly to the mechanisms underlying the neuroprotective action of Semax in the brain under tMCAO conditions. Our study of the neuroprotective properties of Semax was limited to the analysis of the expression of several proteins. Further in-depth studies of the effect of the peptide on the molecular pathways of stroke at the proteome level will definitely clarify the mechanism underlying the neuroprotection afforded by Semax.

## Data Availability

Publicly available datasets were analyzed in this study. This data can be found here: [61,62].

## Supplementary Materials

The following are available online at https://www.mdpi.com/article/10.3390/ijms22126179/s1.
