# Supplementary material for: Brain Protein Expression Profile Confirms the Protective Effect of the ACTH(4–7)PGP Peptide (Semax) in a Rat Model of Cerebral Ischemia–Reperfusion
Source: Int J Mol Sci. 2021 Jun 8;22(12):6179. doi: 10.3390/ijms22126179 (PMC8226508; doi:10.3390/ijms22126179)
Supplement: Supplementary file 1 [file ijms-22-06179-s001.zip › Supplementary Figure S1.pdf]

Supplementary Figure S1. Experimental design.

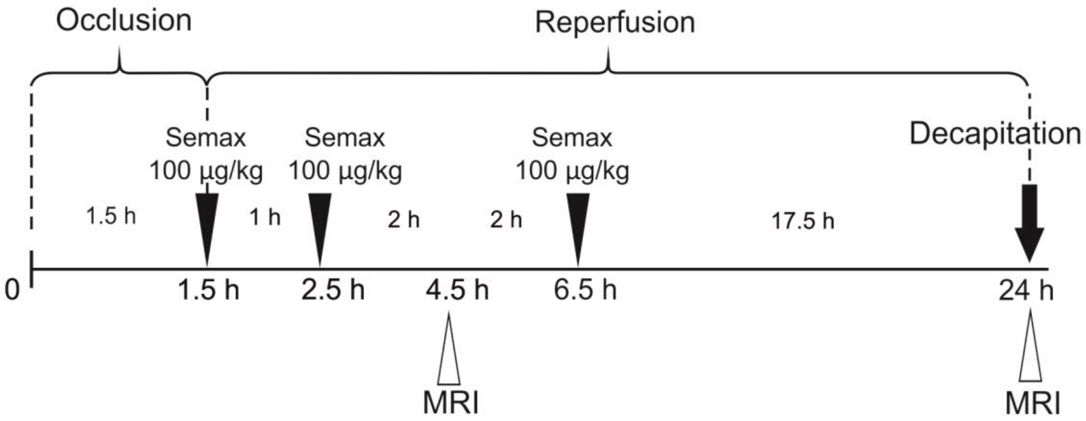

In the rat tMCAO model, the time intervals between the onset of occlusion, reperfusion, Semax injections, MRI diagnostics, and decapitation of animals are indicated.
