# Supplementary material for: Brain Protein Expression Profile Confirms the Protective Effect of the ACTH(4–7)PGP Peptide (Semax) in a Rat Model of Cerebral Ischemia–Reperfusion
Source: Int J Mol Sci. 2021 Jun 8;22(12):6179. doi: 10.3390/ijms22126179 (PMC8226508; doi:10.3390/ijms22126179)
Supplement: Supplementary file 1 [file ijms-22-06179-s001.zip › Supplementary Figure S2.pdf]

Supplementary Figure S2. MRI of ischemic foci after tMCAO.

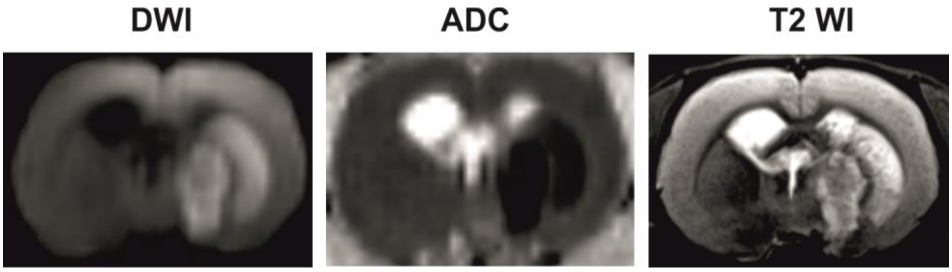

DWI with an ADC map and T2 WI scans of the formation of ischemic injury areas with a subcortical localization in the brain of rats at 24 h after tMCAO.
