# Supplementary material for: Brain Protein Expression Profile Confirms the Protective Effect of the ACTH(4–7)PGP Peptide (Semax) in a Rat Model of Cerebral Ischemia–Reperfusion
Source: Int J Mol Sci. 2021 Jun 8;22(12):6179. doi: 10.3390/ijms22126179 (PMC8226508; doi:10.3390/ijms22126179)
Supplement: Supplementary file 1 [file ijms-22-06179-s001.zip › Supplementary Figure S3.pdf]

**Supplementary Figure S3. The complete raw images of the blots**

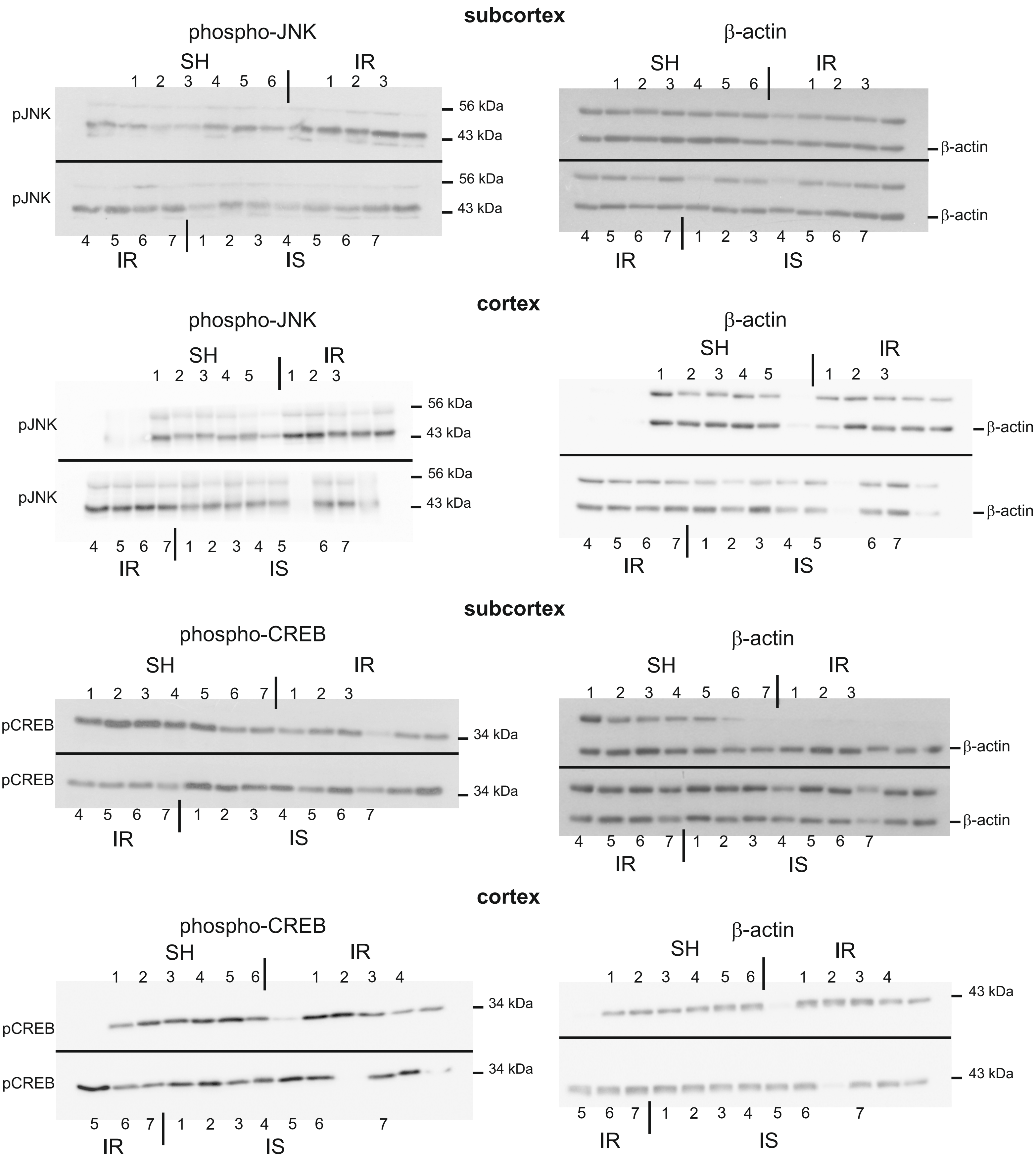


Original immunoblots of active JNK and CREB in the subcortex and cortex in SH, IR and IS groups (n = 5-7) are shown to the left. Corresponding immunoblots of β-actin are shown to the right. The numbers indicate the lane numbers in each group that were used for the semi-quantitative analysis.

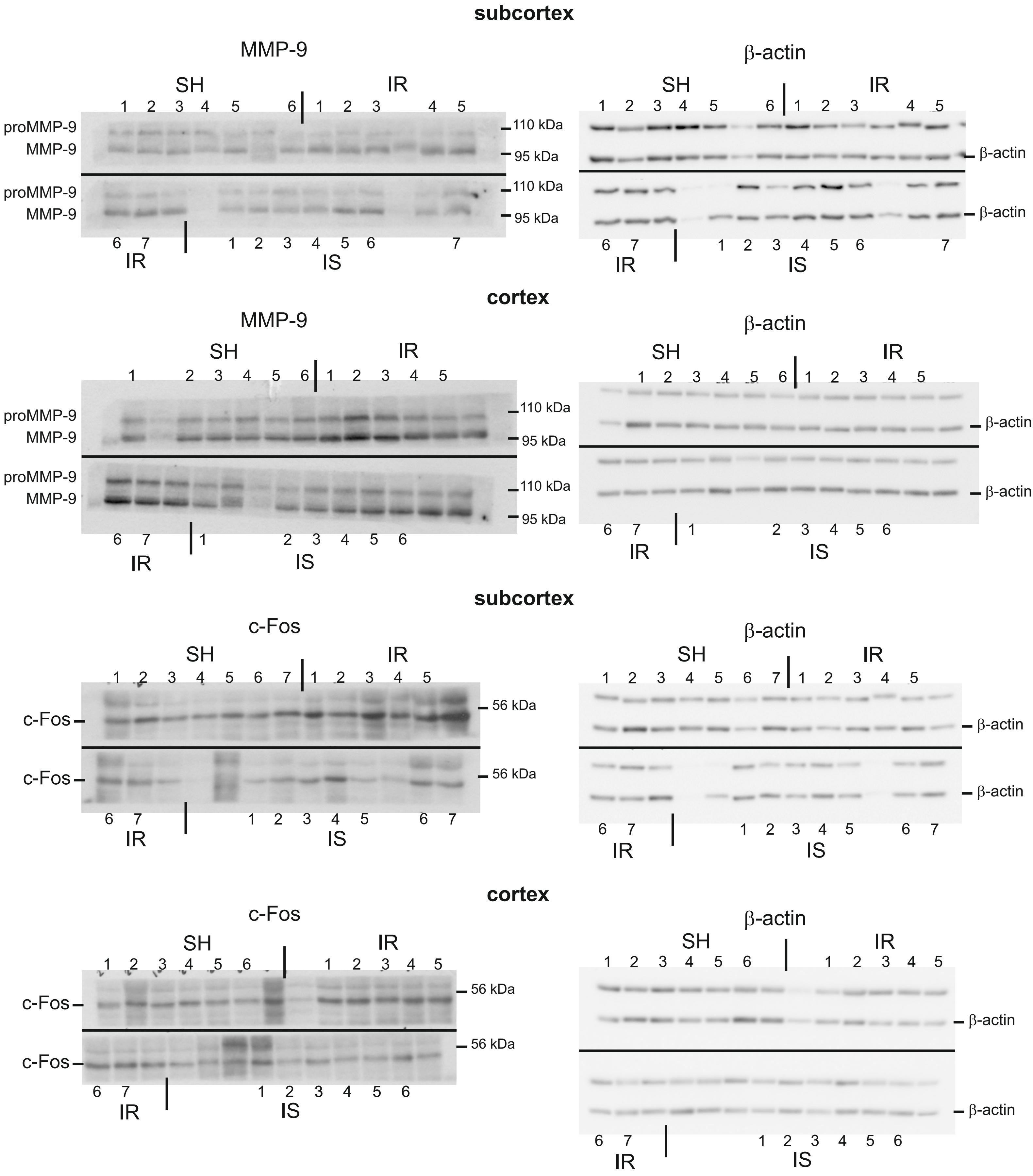


Original immunoblots of MMP-9 and c-Fos in the subcortex and cortex in SH, IR and IS groups (n = 5-7) are shown to the left. Corresponding immunoblots of β-actin are shown to the right. The numbers indicate the lane numbers in each group that were used for the semi-quantitative analysis.
